# Hardware Trojan Attacks on the Reconfigurable Interconnections of Field-Programmable Gate Array-Based Convolutional Neural Network Accelerators and a Physically Unclonable Function-Based Countermeasure Detection Technique

**DOI:** 10.3390/mi15010149

**Published:** 2024-01-19

**Authors:** Jia Hou, Zichu Liu, Zepeng Yang, Chen Yang

**Affiliations:** School of Microelectronics, Xi’an Jiaotong University, Xi’an 710049, China; hjwst0314@stu.xjtu.edu.cn (J.H.); daolzc199947@stu.xjtu.edu.cn (Z.L.); yangzepeng@stu.xjtu.edu.cn (Z.Y.)

**Keywords:** convolutional neural network, reconfigurable CNN accelerator, hardware Trojan, physical unclonable function, field-programmable gate array (FPGA)

## Abstract

Convolutional neural networks (CNNs) have demonstrated significant superiority in modern artificial intelligence (AI) applications. To accelerate the inference process of CNNs, reconfigurable CNN accelerators that support diverse networks are widely employed for AI systems. Given the ubiquitous deployment of these AI systems, there is a growing concern regarding the security of CNN accelerators and the potential attacks they may face, including hardware Trojans. This paper proposes a hardware Trojan designed to attack a crucial component of FPGA-based CNN accelerators: the reconfigurable interconnection network. Specifically, the hardware Trojan alters the data paths during activation, resulting in incorrect connections in the arithmetic circuit and consequently causing erroneous convolutional computations. To address this issue, the paper introduces a novel detection technique based on physically unclonable functions (PUFs) to safeguard the reconfigurable interconnection network against hardware Trojan attacks. Experimental results demonstrate that by incorporating a mere 0.27% hardware overhead to the accelerator, the proposed hardware Trojan can degrade the inference accuracy of popular neural network architectures, including LeNet, AlexNet, and VGG, by a significant range of 8.93% to 86.20%. The implemented arbiter-PUF circuit on a Xilinx Zynq XC7Z100 platform successfully detects the presence and location of hardware Trojans in a reconfigurable interconnection network. This research highlights the vulnerability of reconfigurable CNN accelerators to hardware Trojan attacks and proposes a promising detection technique to mitigate potential security risks. The findings underscore the importance of addressing hardware security concerns in the design and deployment of AI systems utilizing FPGA-based CNN accelerators.

## 1. Introduction

**Figure 1:**
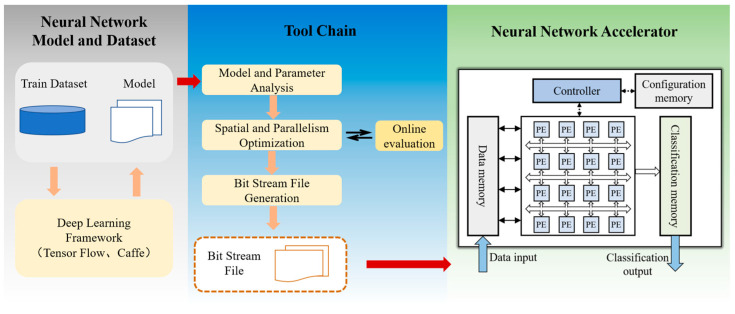
Typical CNN model and reconfigurable accelerator.

Nowadays, convolutional neural networks (CNNs) have become popular for a wide range of tasks, including medical diagnosis [1,2], stock market prediction [3,4], facial recognition [5,6], and semantic segmentation [7,8]. The computational requirements of CNNs can be significant, with hundreds of megabytes of weights and billions of operations needed per inference. To accelerate CNN processing, CNN accelerators have been increasingly utilized to improve inference speed. These accelerators are designed on various platforms, such as central processing units (CPUs), graphics processing units (GPUs), application-specific integrated circuits (ASICs), and field-programmable gate array (FPGAs). One promising approach is the use of dynamically reconfigurable computing architecture [9], which offers both high energy efficiency and flexibility. As a result, numerous reconfigurable CNN accelerators [10,11,12,13,14,15] have been proposed to accelerate different CNN models. Figure 1 provides a high-level overview of a typical accelerator design, which includes a neural network model, a toolchain, and a CNN accelerator. CNN models are trained using extensive datasets to generate weights, while the toolchain translates these models into executable instructions for the accelerator [16]. CNN accelerators effectively utilize hardware resources to perform rapid model inference. As CNN accelerators are widely deployed in AI systems, the security of these accelerators is of the utmost importance for the overall system. Previous research has shown that CNNs are not as robust as desired. Previous studies on CNN security threats have primarily focused on datasets and models, exploring areas such as adversarial examples [17], data poisoning [18], and fault injection [19]. Moreover, security threats associated with CNN accelerators have extended into the hardware domain. Prior works have introduced various hardware attack methods, including reverse engineering [20] and hardware Trojans [21,22,23,24,25,26,27]. Among these threats, hardware Trojan attacks are considered a major security concern [28,29,30]. Integrated circuits (ICs), including those used for designing and manufacturing CNN accelerators, increasingly rely on untrusted parties throughout their lifetime, including unverified individuals, design tools, third-party intellectual property (IP), and components [31]. Such a complex supply chain in IC design houses poses significant challenges in maintaining control over the entire process, from design to manufacture. This vulnerability opens up opportunities for malicious manipulations and compromises. Additionally, FPGA, being the main hardware platform, is not immune to hardware Trojan attacks. In the context of FPGA, hardware Trojans can be classified into three types: Trojans in FPGA fabric, Trojans in the FPGA toolchain, and Trojans in the FPGA bitstream [32]. Trojans in FPGA fabric can be inserted during fabrication at an untrusted foundry or during the design phase by a rogue employee. These Trojans have the ability to add or delete gates to conduct malicious activities or modify device parameters to compromise the reliability of the FPGA [33,34]. The threat posed by design tools to hardware designs has long been recognized [35,36,37]. Design compromise can occur either due to compromised design tools, where attackers include malicious code that is compiled into a binary version of the tools, or through collaboration between design tool vendors and intellectual property (IP) vendors, where malicious behavior is embedded into the IP cores during synthesis. Hardware Trojans can also be inserted by modifying the FPGA configuration bitstream, and this can be conducted in two ways: Type-I Trojans and Type-II Trojans. Type-I Trojans are defined as Trojan circuits that do not overlap with the original circuit in terms of resources and area. In contrast, Type-II Trojan circuits are interconnected with the original circuits, resulting in resource overlap within the FPGA. It is important to note that even a small disturbance caused by a hardware Trojan can lead to significant reductions in accuracy or unexpected classification results for CNNs. Therefore, attacks through hardware Trojans pose significant challenges for FPGA-based CNN accelerators and have the potential to cause severe damage to AI systems.

In the current landscape, several hardware Trojans have been specifically designed to target CNN accelerators [21,22,23,24,25,26,27]. These attacks primarily focus on the process elements (PEs) [21,22], memory [23,24], prediction results [25], bias buffer [26], and activation parameters [27], but they often overlook the reconfigurable interconnection network, which is another vital component. As depicted in Figure 1, a typical reconfigurable accelerator consists of a PE array that can be dynamically configured to perform convolution operations in different computation modes. The configuration of the PE array relies on the reconfigurable interconnection network to adjust data paths. Therefore, the reconfigurable interconnection network plays a crucial role in ensuring accurate computations of CNN models. Consequently, attacking the reconfigurable interconnection network through hardware Trojans is a noteworthy consideration. To address this issue, countermeasures must be taken to protect the reconfigurable interconnection network. This paper proposes a hardware Trojan that specifically targets the dynamically reconfigurable interconnection network between PEs on CNN accelerators. Furthermore, to defend against hardware Trojan attacks and safeguard the reconfigurable interconnection network, a countermeasure based on physically unclonable functions (PUFs) is introduced. The main contributions of this paper can be summarized as follows:

(1) An attack method is designed to exploit a hardware Trojan on the reconfigurable interconnection network of an FPGA-based CNN accelerator. The objective of this attack is to manipulate the data path once the hardware Trojan is activated. Through this manipulation, the consequences become evident, as the PE array becomes incapable of performing the required calculations.
(2) A detection technique based on physical unclonable functions (PUFs) is proposed as a countermeasure to protect the reconfigurable interconnection network of an FPGA-based CNN accelerator against hardware Trojan attacks. The designed PUF serves a dual purpose: it can detect the presence of hardware Trojans and identify their specific locations within the system.
(3) The implemented hardware Trojan is embedded within the interconnection network of an in-house reconfigurable CNN accelerator. To evaluate its effectiveness, the Trojan attack was carried out on popular CNN architectures such as LeNet, AlexNet, and VGG. The experimental results reveal a significant degradation in accuracy for the CNN accelerator, ranging from 8.93% to 86.20% once the Trojan is activated. Furthermore, a detection technique based on an arbiter-based PUF is implemented on a Xilinx Zynq XC7Z100 platform. This PUF serves to detect hardware Trojans within the reconfigurable interconnection network. The effectiveness of the proposed detection technique is validated through these experimental implementations.

Our paper consists of five sections. Section 2 provides an introduction to the related work focusing on significant threats and the motivation behind studying the security issue. This section is divided into two subsections. The Section 2.1 highlights prior works related to hardware Trojan attacks on traditional ICs, various threats to CNN models, and attack methods for CNN accelerators. Section 2.2 specifically discusses the main motivations that drove us to study the security issue for FPGA-based CNN accelerators. In Section 3, we delve into the methods of hardware Trojan attacks on reconfigurable interconnection networks, along with the corresponding countermeasure utilizing PUF-based detection techniques. Section 3.1 presents the structure of the reconfigurable interconnection network and the threat model concerning the hardware Trojan targeting this network. Section 3.2 introduces the arbiter-based PUF and its application as a countermeasure against hardware Trojan attacks. Section 4 focuses on presenting the evaluation results for both the hardware Trojan attacks and the PUF-based detection technique. Section 4.1 provides insights into the experimental setup, including the utilization of an in-house CNN accelerator, an FPGA platform, CNN models, and datasets. Section 4.2 offers the evaluation results of the hardware Trojan attack from three key aspects. Section 4.3 presents the detection results for the PUF-based detection technique. Finally, the Section 5 discusses the entirety of our work and outlines possible future directions for research and development.

## 2. Related Work and Motivation

### 2.1. Related Work

The significant threat posed by hardware Trojan attacks in the field of integrated circuits (ICs) and their wide application in the field of artificial intelligence (AI) have prompted extensive research efforts.

Advancements in the understanding and mitigation of hardware Trojan attacks have been made since the seminal paper on hardware Trojans in 2007 [38]. A comprehensive overview of research on hardware Trojans was provided in [39,40], which discussed the potential threats posed by hardware Trojans, presented attack models, and explored countermeasures against such attacks. Novel triggering techniques, such as utilizing do not-care states in designs, were introduced [41]. MOLES [42] proposed the use of new payloads that could generate intentional side-channel signals to leak secret information. To evade detection, a circuit with gate resizing was redesigned at minimal cost without impacting the circuit’s functionality [43]. Zero overhead malicious modifications were proposed for high-performance and embedded microprocessors, where hardware Trojans were activated under specific conditions to obfuscate modifications [44]. Regarding FPGAs, hardware Trojans inserted via software-based bitstream modifications were discussed, with a focus on unencrypted bitstreams [45]. The lack of verification mechanisms for bitstream file correctness makes this type of modification particularly challenging to detect. Additionally, an attack targeting FPGA design tools was presented, demonstrating the injection of malicious hardware Trojans into designs during synthesis, thereby evading certain hardware Trojan detection techniques [35]. Side-channel analysis techniques were utilized to detect malicious inclusions that affect power consumption [46]. Security monitors embedded within ICs during the design phase were introduced to detect unexpected or illegal behavior caused by Trojans [47]. This approach complements other detection methods. Furthermore, a real-time online learning approach [48] was proposed to proactively prevent unforeseen attacks. This adaptive approach, based on machine learning, enables the real-time detection of new attacks as they emerge, thereby enhancing the security of designs.

For CNNs, several studies [17,18,19] have focused on attacking the models and training data. Adversarial examples were designed to deceive deep learning models, and existing defense mechanisms were discussed [17]. Data poisoning attacks were devised to manipulate forecasting models in markets for malicious gains [18]. Another approach involved modifying the parameters of a DNN through fault injection to misclassify specific input patterns into adversarial classes [19]. More recently, attacks targeting the hardware side of CNNs have emerged. A reverse engineering attack leveraging side-channel information leaks was proposed to infer the underlying network structure, even in the presence of data encryption [20]. The first study to explore the hardware Trojan threat on CNN-based image classification inserted a Trojan into a Multiplier and Adder Tree (MAT) module of an FPGA accelerator [21]. The experimental results demonstrated precise control over CNN classification results with minimal overhead and modification. Another novel technique injected a hardware Trojan into a rectified linear unit (ReLU) function block, achieving adversarial goals [22]. The experimental results showcased the stealthiness and effectiveness of these injected hardware Trojans. Different from previous approaches that required knowledge of both the model and toolchain, a memory Trojan was designed for a neural network accelerator. This Trojan performed an accuracy degradation attack by accessing memory bus data, effectively activated by specific trigger images [23]. A sequence-triggered hardware Trojan was proposed to control the prediction results of neural network systems. This method relied on a sequence of input images rather than modifying individual pixels, making it robust to image pre-processing and imperceptible to human observers [25]. Experiments conducted on MNIST, CIFAR100, and ISLVRC datasets demonstrated the Trojan’s capability to activate selectively with minimal hardware overhead. Int-Monitor, a hardware Trojan targeting DNN accelerators, was designed to attack the global bias buffer. By implanting an interrupt monitor between the host processor and the DNN accelerator, this Trojan prevented the activation of neurons in a DNN model, rendering the network’s forward propagation invalid and the accelerator unresponsive [26]. Runtime experiments on various DNN models showed the successful exploitation of FPGA-based DNN accelerator SoCs by Int-Monitor. The Trojan incurred minimal hardware overhead and negligible power consumption, with average hardware overhead of 0.5% and 0.2% and power consumption of 0.622% and 0.187% in SIMD and NVDLA accelerators, respectively [26]. Novel hardware Trojan attacks specifically targeting DNN hardware accelerators implemented on FPGA were introduced [27]. These hardware Trojans aim to modify the activation parameters of the DNN within the accelerator. The experimental results illustrate that the proposed hardware Trojan attacks exhibit high levels of stealthiness, making them difficult to detect. When activated, these Trojans lead to substantial degradation in the accuracy of the DNN in terms of its inference capabilities.

### 2.2. Motivation

There are two main motivations that drive us to study this security issue for FPGA-based CNN accelerators.

**Figure 2:**
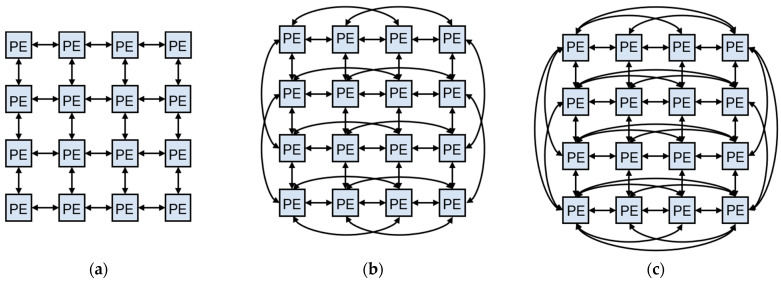
Three typical reconfigurable interconnection networks: (**a**) MESH structure; (**b**) MESH_PLUS structure; (**c**) MORPHOSYS structure.

Firstly, there is a noticeable gap in research focusing on hardware attacks specifically targeting CNN accelerators. Existing hardware attacks have primarily focused on process elements (PEs), memory, and other components of CNN accelerators [21,22,23]. However, there is a lack of studies investigating hardware attacks on the reconfigurable interconnection network. This network plays a crucial role in achieving accurate and efficient inferences in accelerators. Its function is to facilitate communication between PEs by connecting their data paths. The structure of the reconfigurable interconnection network is not fixed and can vary depending on the requirements of the application. It needs to strike a balance between flexibility and efficiency. Accelerators designed with this interconnection concept can leverage the advantages of reconfigurable computing architecture, such as low power consumption, high performance, and extensibility. Figure 2 illustrates three common reconfigurable interconnection network architectures used in reconfigurable processors. The MESH structure (Figure 2a) connects adjacent PEs and enables efficient execution of complex operations like matrix multiplication and inverse. The MESH_PLUS structure (Figure 2b) and the MORPHOSYS structure (Figure 2c) are extensions of the MESH structure that offer more flexible connections, allowing for a greater variety of operator mappings. In any type of reconfigurable interconnection network, the implementation relies on multiplexers (MUXs). MUXs select the appropriate data paths based on control signals. Reconfigurable CNN accelerators, such as Eyeriss [10], Thinker [11], and others [13,14,15], employ MUXs to construct their interconnection networks. It is evident that the reconfigurable interconnection network, particularly the MUXs, is a fundamental module for achieving flexibility in reconfigurable CNN accelerators. MUXs ensure that the PE array can be configured to perform accurate computations. Therefore, hardware Trojan attacks targeting the reconfigurable interconnection network, especially the MUXs, can have detrimental effects on the prediction accuracy of CNN accelerators.

**Figure 3:**
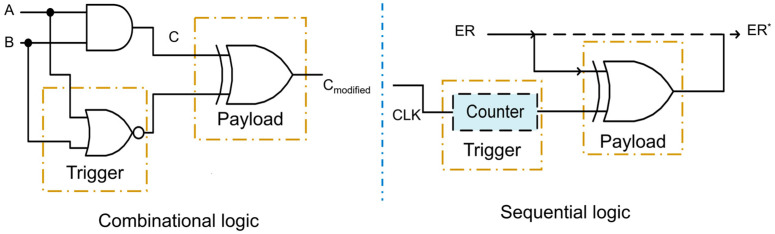
Typical structure of hardware Trojan.

Secondly, the motivation for studying security issues in CNN accelerators is the scarcity of targeted countermeasures against hardware attacks. CNN accelerators play a critical role as decision makers in various AI systems. Therefore, once these accelerators become compromised, significant damage can be inflicted on the entire AI system. Traditional hardware attack methods can be adapted to target CNN accelerators, posing a serious threat to their security. Hardware Trojan attacks, in particular, are highly concerning. A hardware Trojan is a malicious design component that is covertly inserted into original circuits and can evade detection during conventional post-manufacturing tests. When triggered under specific conditions, the Trojan can leak sensitive information or manipulate the logic functions of the system. Hardware Trojan attacks can be categorized into combinational logic and sequential logic, depending on the type of circuits involved. Figure 3 illustrates the structure of a hardware Trojan, consisting of a trigger and a payload. In Figure 3, A, B, C are input signals and superscript * indicates the modified signal. The trigger activates the payload under rare conditions, allowing it to modify the original values. Various Trojan detection technologies have been proposed to mitigate the threats posed by hardware Trojans, but no single method can detect and eliminate all Trojans and their associated hazards [49,50]. Furthermore, existing studies lack adequate protection against hardware Trojan attacks specifically targeting CNN accelerators. Therefore, exploring countermeasures against hardware Trojan attacks is crucial. PUFs [51] are functional circuits that rely on physical quantities, such as distance, time, and direction. PUFs leverage inherent process variations during manufacturing to achieve the unique functionality of generating a challenge–response pair (CRP) that is practically impossible to replicate in another device. PUFs have been utilized in various applications, including hardware-based security, anti-counterfeiting measures, and secure key storage. They offer a means to enhance device security by leveraging natural variations in the manufacturing process, making it difficult for attackers to replicate or forge device identities. Taking advantage of PUF techniques, this paper proposes a detection technique to protect the reconfigurable interconnection network from hardware Trojan attacks. This detection method can also be applied to safeguard similar structures with reconfigurable interconnection networks in CNN accelerators. By integrating PUF-based protection, the security of CNN accelerators can be enhanced against hardware Trojan threats, ensuring the reliability and trustworthiness of these critical components in AI systems.

## 3. Methods

### 3.1. Hardware Trojan against Reconfigurable Interconnection Network

In this subsection, an in-house CNN accelerator with a reconfigurable interconnection network is presented as the foundation for a hardware Trojan attack model. By analyzing and targeting the interconnection network, the proposed hardware Trojan attack models can be deployed against an in-house CNN accelerator [52]. The design of this reconfigurable interconnection network is not specific to a particular CNN accelerator but is applicable to most CNN accelerators. As a result, the proposed hardware Trojan attack model can be adapted to target other CNN accelerators with similar interconnection structures.

#### 3.1.1. FPGA-Based CNN Accelerator and Reconfigurable Interconnection Network

**Figure 4:**
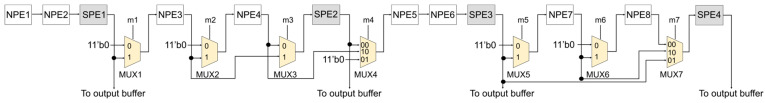
The reconfigurable interconnection of a dynamically reconfigurable accelerator.

An FPGA-based CNN accelerator named RNA [12,53] features a reconfigurable interconnection structure designed specifically for accelerating neural network inference. This particular interconnection structure is commonly found in dynamic accelerators like Eyeriss [10], Thinker [11], and other similar accelerators [13,14,15]. Figure 4 illustrates the architecture of the RNA accelerator. It comprises Normal Processing Elements (NPEs), which are responsible for carrying out multiplication and addition operations, and Special Processing Elements (SPEs), which additionally incorporate an activation function. When the activation function is not required, the SPEs can be switched to function as NPEs. The selection values of multiplexers (MUXs), denoted as m1–m7, control the data paths within the accelerator. By manipulating these MUXs, the PE array can be dynamically configured to support three computation modes, representing kernel sizes of 3 × 3, 5 × 5, and 11 × 11. Table 1 outlines the configuration words necessary for performing the three different kernel sizes mentioned above. To illustrate, consider the 3 × 3 kernel size mode. According to the configuration words for the first row in Figure 3, every two NPEs and one SPE form a subset within the PE array. In this case, m1, m3, and m5 are set to 0, while m2 and m6 are set to 1. Furthermore, m4 is set to 01, and m7 is set to 00. As a result, the PE array can be partitioned into four subsets for performing convolutional operations: NPE1, NPE2, and SPE1; NPE3, NPE4, and SPE2; NPE5, NPE6, and SPE3; and NPE7, NPE8, and SPE4. The reconfigurable interconnection network plays a crucial role in ensuring the correct computation of the PE array. Consequently, hardware Trojan attacks targeting this component can have detrimental effects on the prediction accuracy of CNN accelerators.

#### 3.1.2. The Presence of Hardware Trojans in the Interconnection Network

Dynamically reconfigurable computing architectures have the potential to achieve high energy efficiency and flexibility [9]. As a result, several reconfigurable CNN accelerators, such as Eyeriss [10], Thinker [11], and RNA [12,53], have been proposed to accelerate various CNN models. These accelerators share a common structure, featuring a PE (Processing Element) array that can be dynamically configured to perform convolution operations based on specific computation modes. The configuration of the PE array relies on the reconfigurable interconnection network, which plays a crucial role in adjusting data paths. Consequently, the interconnection network is a critical component of CNN accelerators. In contrast to previous Trojan attacks that targeted PEs and memory, the proposed hardware Trojan specifically attacks the reconfigurable interconnection network in CNN accelerators. The attack consists of two phases: the triggering phase and the payload phase. In the triggering phase, there are two possible triggering conditions: configuration words and input images. In the first case, the configuration words determine the computation mode of the PE array. The configuration words serve as the triggering condition, meaning that the Trojan will be activated whenever the computation mode related to the configuration words is used. Consequently, this can lead to severe damage to the accelerators. In the second case, the trigger relies on input images. The adversary can choose specific bits of the input images to act as the triggering condition. Ordinary images will not trigger the payload, allowing the adversary to selectively initiate the attack against the accelerators. This provides the adversary with control over the attack process. During the payload phase, once the Trojan is activated, it maliciously modifies the connections between the PEs. This results in the misconfiguration of the computational circuit, thereby achieving the objective of an accuracy degradation attack.

#### 3.1.3. Threat Model for Hardware Trojans against the Interconnection Network

**Figure 5:**
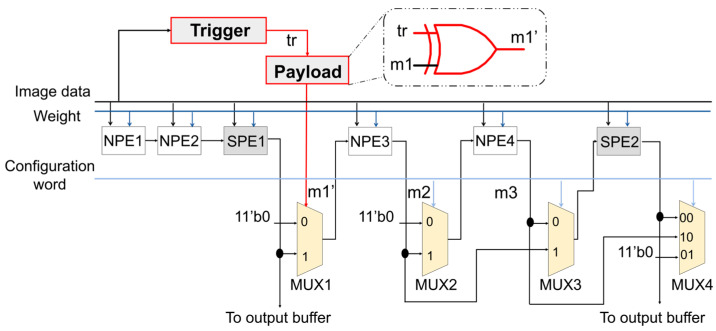
Hardware Trojan insertion at the MUX1.

In our work, hardware Trojans are specifically implemented in a crucial component of CNN accelerators: the reconfigurable interconnection network. Figure 5 illustrates how a hardware Trojan modifies the selection value of MUX1 (m1) in this interconnection network. The trigger for this Trojan utilizes input images as the triggering condition, and the payload is designed with an exclusive or (XOR) gate to corrupt, when triggered, an internal signal. To demonstrate the impact of a hardware Trojan against CNN accelerators, we consider the example of performing convolution computation with a 3 × 3 kernel size. Theoretically, signal m1 should have a value of 0 when the kernel size is 3 × 3. However, when the Trojan is activated (i.e., tr signal is 1), signal m1 is modified to its opposite, m1′ under the action of the XOR gate. This modification causes the output of SPE1 to be connected with the input of NPE3. In other words, it introduces the output result of the first subset of PEs (NPE1, NPE2, and SPE1) into the second subset (NPE3, NPE4, and SPE2). As a result, the second subset of PEs generates an incorrect output value. Consequently, using this subset of PEs for convolutional computation leads to incorrect results. It is worth mentioning that hardware Trojans can also be connected to other MUXs, causing different types of attack consequences.

Table 2 lists potential attack consequences that can arise from hardware Trojans at different positions within the interconnection structure shown in Figure 4. The table defines various variables: N represents the output results of the NPE element, SP represents the output results of the SPE element, S represents the normal result of a subset, and S’ represents the questionable result after modification due to a Trojan attack. To illustrate the attack consequences described in Table 2, let us consider two examples. In the first case, we observe that S’2 is equal to S2 minus N3 and N4 (i.e., the first row, third column). In a normal scenario where no Trojan is activated or present, the output result of the second subset should be S2. However, when the Trojan is activated, the actual output result becomes S’2, which equals S2 minus N3 and N4. This alteration means that the result after the malicious modification no longer contains the output of NPE3 and NPE4. In terms of convolutional computation, this attack consequence signifies that the first two lines of the convolution kernel and the input image are not computed correctly. Another case mentioned is S’2 being equal to S2 plus S1 (i.e., the first row, first column). This case aligns with the depiction in Figure 5, wherein the output of a PE subset is additionally added to other output results. These two cases represented here are examples of the different cases listed in Table 2. In essence, hardware Trojan attacks on the reconfigurable interconnection network can lead to either overlapping adjacent image data blocks or the exclusion of pixels from an image data block during convolutional computing. Consequently, these attack scenarios indicate that the proposed hardware Trojan attack can result in incorrect convolutional computation, leading to degradation in terms of accelerator performance.

### 3.2. Corresponding Countermeasure of PUF-Based Detection Technique

PUF is a functional circuit that relies on various physical quantities such as distance, time, and direction. It takes advantage of unescapable process errors that occur during the manufacturing process to achieve the unique function of a challenge–response pair (CRP). This uniqueness makes it practically impossible to construct an object with an identical structure [51]. This inherent uniqueness provides a high level of security and resilience against unauthorized replication or tampering attempts.

Taking advantage of the unique properties of PUF, the PUF-based countermeasure has been utilized to safeguard the interconnection network addressed in the paper. By integrating PUFs into the interconnection network, the countermeasure adds an additional layer of protection against hardware Trojan attacks. The unique challenge–response pairs generated by the PUFs can be utilized to authenticate the configurations of the network. This helps in detecting and mitigating any potential hardware Trojans that might be present.

#### 3.2.1. Arbiter-Based PUF

**Figure 6:**
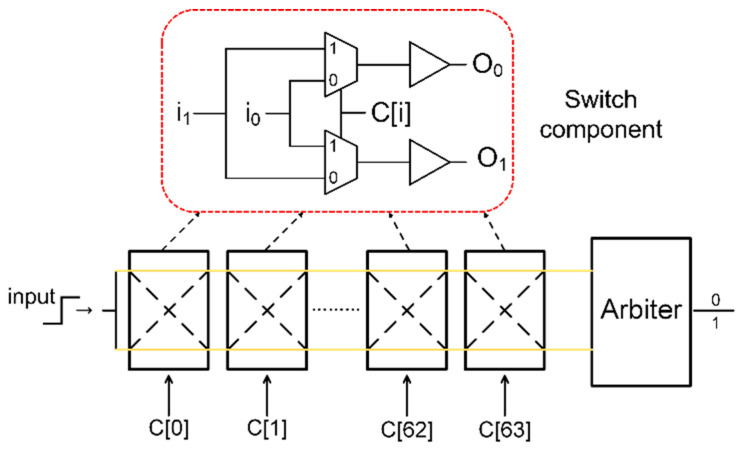
Arbiter-based PUF circuit.

An arbiter-based PUF [54] harnesses the statistical delay variation of wires and transistors across integrated circuits (ICs) to generate CRPs. This type of PUF comprises switch components and an arbiter positioned at the end of delay paths, as illustrated in Figure 6. The PUF consists of 64 switch components, and each switch component is represented by a red dotted line in the figure. Each switch component, implemented with a pair of 2-to-1 multiplexers and buffers, is composed of two input ports (i0 and i1), one control port (C[i]), and two output ports (O0 and O1). The fundamental idea behind this design is that the arbiter determines the speed of signal transmission along two symmetrical delay paths. The circuit takes 64 challenge bits (C[0]~C[63]) as inputs to configure the delay paths and generate a 1-bit response as an output [54]. If the signal traveling through the bottom path arrives first at the arbiter, the output response is set to 1; otherwise, the output response is 0.

#### 3.2.2. PUF-Based Countermeasure against Hardware Trojans Attack

A dynamically reconfigurable chip combines the benefits of high performance in static situations, similar to ASICs, with the dynamic configurability necessary to meet the diverse requirements of different applications in dynamic scenarios.

**Figure 7:**
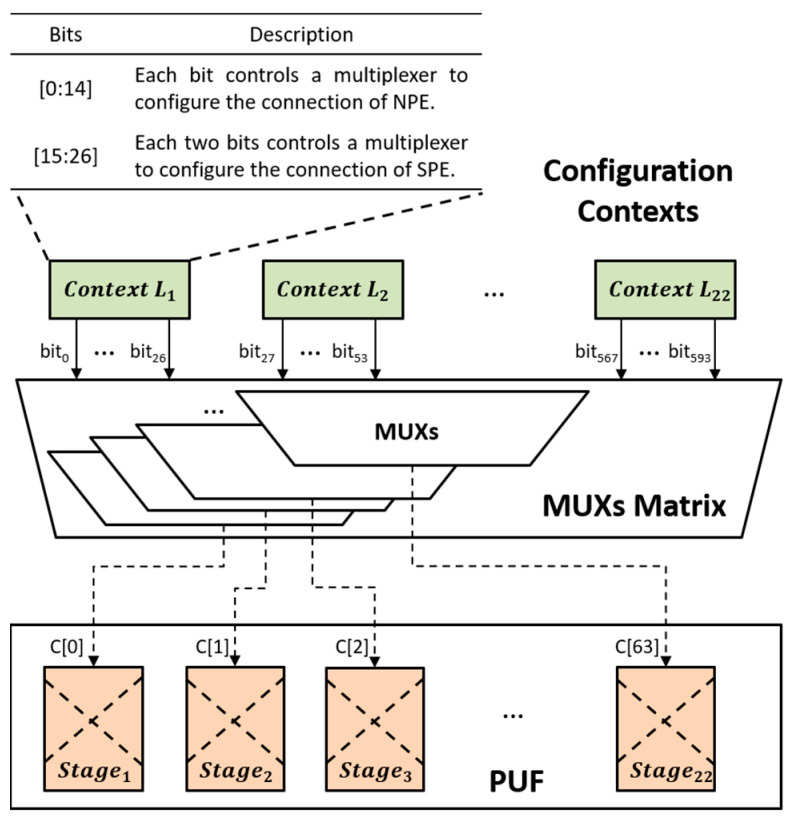
The map of configuration context on MUXs matrix.

In the case of a hardware Trojan in a reconfigurable interconnection network, its impact is observed in the modification of control bits for the multiplexers (MUXs) among processing elements (PEs), specifically the configuration bits that influence the interconnection network. Once triggered, the hardware Trojan alters the interconnecting structure between PEs, leading to disruptions in data transmission and potentially causing calculation errors. To detect hardware Trojans in a reconfigurable interconnection network, a mapping technique can be employed. Figure 7 showcases the mapping of configuration contexts to an arbiter-based PUF. In this configuration, all the configuration contexts for 22 lines of PEs (represented as Context L1, Context L2, …, and Context L22) are connected to 64 challenge ports (C[0]~C[63]) through a MUX matrix. The matrix consists of 64 MUXs, each responsible for selecting one bit out of the nine or ten configuration bits.

**Figure 8:**
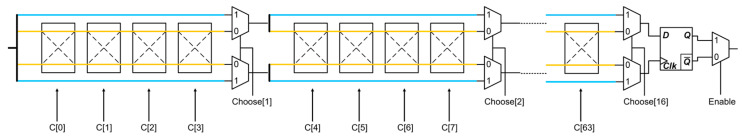
The framework of PUF-based hardware Trojan detection technique.

In order to defend against the proposed attack, a PUF-based countermeasure is designed, as shown in Figure 8. This countermeasure leverages the dynamic configurability characteristic of the reconfigurable chip to configure an arbiter-PUF using 2-to-1 multiplexers (MUXs) in the MUX matrix. The proposed design extends a traditional arbiter-based PUF by adding an additional 2-to-1 MUX to select the final output. By enabling this MUX, the countermeasure increases the possible output variations, making it challenging for an attacker to predict or crack the response and thereby enhancing the security level. Furthermore, all the switch components are divided into 16 sets, with each set containing 4 switch components. At the end of each set, a pair of 2-to-1 MUXs is employed to determine whether the current set is involved in the PUF. This configuration allows for flexible configuration of the delay paths. When the control bit choose[i] is set to 1, the delay paths bypass the current set, meaning that the input signal does not pass through the paths of that set. Conversely, for choose[i] = 0, the input signal traverses the delay path of that set. By utilizing this countermeasure design with the dynamic configurability of the reconfigurable chip, the circuit can effectively defend against the proposed hardware Trojan attack in the interconnection network, enhancing security and preventing potential disruptions in data transmission.

**Figure 9:**
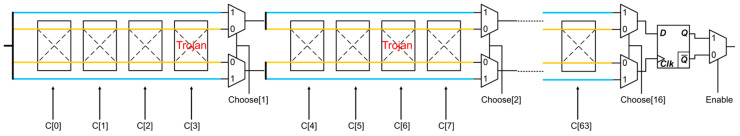
Hardware Trojans in two sets.

The PUF-based countermeasure offers two effective functions: detecting the presence of hardware Trojans and locating their positions within the interconnection network. For the first function, when hardware Trojans modify the control bit (C[i]) of the MUXs, the resulting delay path will differ from that without the modification. By applying constant challenges to the input signal of the PUF, a set of responses can be obtained. To detect hardware Trojans, these resulting response sets are compared with the original set. If there is a significant difference, it indicates the presence of hardware Trojans. For the second function, let us consider a scenario where the hardware Trojan modifies C[3] by placing a switch component. Under choose[1] = 1 (skipping the first set), the challenge–response pairs (CRPs) remain the same as the original CRPs. However, under choose[1] = 0, the CRPs differ from the original. This provides a clue to locate the position of the Trojan in the first set. Similarly, if hardware Trojans exist in multiple sets, a comparison of CRPs under different configurations can help identify the affected sets. Table 3 illustrates the possible results for Trojans in two sets in Figure 9. To summarize the process, when comparing CRPs under different cases, mismatches indicate the existence of Trojans within the corresponding sets. By controlling the choose signals and analyzing the CRP differences, it becomes possible to detect Trojans and pinpoint their specific positions within the interconnection network. This method remains valid for detecting Trojans in multiple sets by adjusting the choose signals and comparing the CRPs accordingly.

## 4. Results

### 4.1. Experimental Setup

To evaluate the proposed hardware Trojan design, the work utilized an in-house reconfigurable CNN accelerator called RNA [51]. The RNA accelerator is implemented on a Xilinx Zynq XC7Z100 platform, which provides both programmable logic and processing capabilities. This accelerator is designed to support three popular network models: LeNet, AlexNet, and VGGNet. The models were trained to classify different datasets: CIFAR-10 for AlexNet and VGGNet, and MNIST for LeNet. In the evaluation process, configuration words and 24-bit image data were used as the triggering conditions to activate the payload of the hardware Trojan. These conditions were carefully chosen to simulate a real-world scenario in which the Trojan payload would be triggered during the operation of the reconfigurable CNN accelerator. By conducting experiments and analysis on the RNA accelerator with the trained network models and appropriate triggering conditions, the researchers were able to evaluate the impact and effectiveness of the proposed hardware Trojan design in terms of its ability to modify control bits and potentially cause calculation errors in the interconnection network.

### 4.2. Hardware Trojan Attack Evaluation

The proposed hardware Trojan attack is evaluated based on three key aspects: stealthiness, attack effectiveness, and hardware overhead. Stealthiness evaluates how well a hardware Trojan hides its existence from detection methods. The goal of a stealthy Trojan is to remain undetected for as long as possible to either gather information or wait for a specific trigger condition before activating. Evaluating stealthiness helps ensure that the attack mechanisms are designed to operate covertly, minimizing the risk of being detected by security systems or vigilant users. Attack effectiveness is a metric that assesses the impact or damage caused by a hardware Trojan once it is activated. It measures the success of the attack in achieving its specific goals, such as significant accuracy degradation in the system. The evaluation of attack effectiveness helps users understand the potential harm that can be inflicted by hardware Trojan attack mechanisms. By assessing this metric, it becomes possible to gauge the severity and seriousness of an attack and develop appropriate defense strategies to mitigate its impact. For the overhead of hardware Trojan attacks, it is important to keep them as minimal as possible to avoid detection and maintain the functionality and performance of the accelerator. Evaluating the overhead helps us understand the practicality and effectiveness of such attacks and allows for the development of countermeasures to detect and mitigate the risks associated with hardware Trojans. These evaluations aim to provide a comprehensive understanding of the attack’s impact and feasibility.

**Figure 10:**
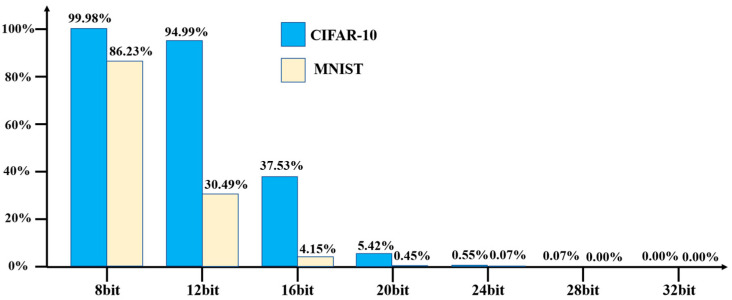
Percentage of original images that trigger hardware Trojans.

Stealthiness is indeed a crucial metric for evaluating attack mechanisms to ensure that triggering conditions stay hidden and evade suspicion from normal users. In the conducted experiment, 50,000 images from the CIFAR-10 and MNIST datasets were used to test the triggering results with different predefined triggering conditions. Figure 10 illustrates the percentage of original images from both datasets that successfully triggered the hardware Trojans. The results indicate that when using eight bits of the original images as the triggering condition, 99.98% and 86.23% of the images from CIFAR-10 and MNIST datasets, respectively, were able to activate the hardware Trojans. When the triggering condition was increased to 12 bits of image data, the proportion of images triggering the Trojans remained high, with 94.99% of CIFAR-10 images being successful triggers. However, the proportion decreased significantly for the MNIST dataset, with only 30.49% of the images triggering the Trojans. Continuing to increase the number of bits in the triggering condition to 16 bits resulted in a further decrease in the activation percentages for both datasets. For the CIFAR-10 dataset, 37.53% of the original images were successful triggers, while for the MNIST dataset, the percentage dropped to 4.15%. Using 20 bits as the triggering condition, the experiments showed a scarcity of images that could activate the hardware Trojans. Only 5.42% of the CIFAR-10 dataset and 0.45% of the MNIST dataset’s original images were successful triggers. Finally, when using 24 bits of image data as the triggering condition, it was observed that almost none of the original images from both datasets were able to activate the hardware Trojan payloads. It is evident from the experimental results that as more bits of image data are used as the triggering condition, it becomes increasingly challenging to trigger the hardware Trojans. Notably, almost no original images were able to trigger the Trojans with 28 or 32 bits of image data.

**Figure 11:**
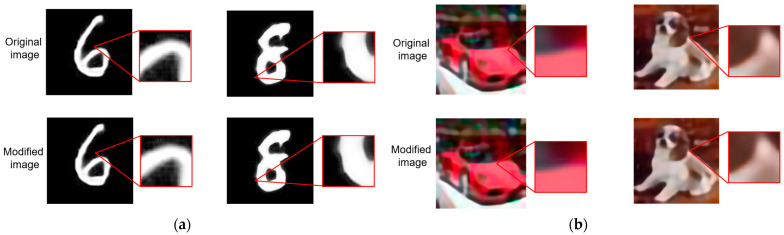
The comparison between original and modified images for different datasets: (**a**) the comparison between original and modified images for MNIST dataset; (**b**) the comparison between original and modified images for CIFAR-10 dataset.

In the experiment, modifications were made to some image data in order to activate the hardware Trojans. To ensure that these modifications would not be easily detectable by humans, a comparison was made between the original images and the modified images using python 3. Figure 11 illustrates this comparison for both the MNIST dataset and the CIFAR-10 dataset. In the case where 20 bits of image data served as the triggering condition, the 20 bits of image data corresponding to the numbers ‘6’ and ‘8’ in the MNIST dataset were modified according to the triggering condition. As shown in Figure 11a, the modified images appear almost identical to the original images, making it difficult for the human eye to detect any differences. This lack of noticeable aberrations helps to evade suspicion. Similarly, in Figure 11b, the comparison between the original and modified images of the CIFAR-10 dataset is shown. For this dataset, the 20-bits data of a ‘car’ and a ‘dog’ were modified, and the differences between the original and modified images are again extremely challenging for humans to discern. These alterations are designed to go unnoticed, thus minimizing the chances of raising suspicion. However, it is crucial to note that even though the modifications are imperceptible to human observers, once these modified images are passed through the accelerator with the hardware Trojans, the Trojans are activated, leading to changes in data paths during computation.

**Figure 12:**
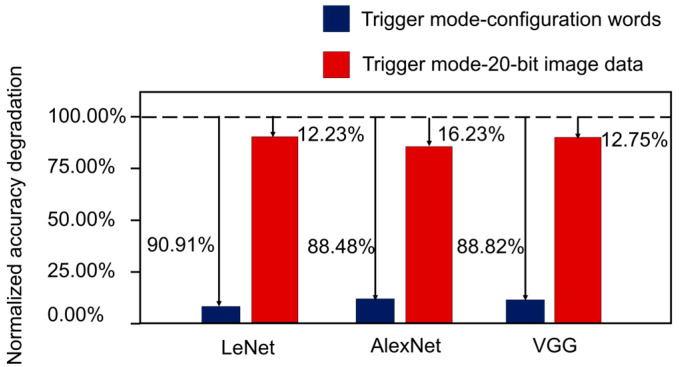
Attack effectiveness of hardware Trojans.

In assessing the attack effectiveness of the designed hardware Trojan attack, the degree of damage to accelerators is evaluated based on accuracy degradation. Table 4 presents the accuracy of the normal mode (no Trojans) and the triggering modes (activated Trojans). Figure 12 visualizes the results of the attack effectiveness, where the accuracy of different modes from Table 4 is normalized with respect to the normal mode. First, the attack effectiveness of using model configuration words as the triggering condition is evaluated. This scenario represents the maximum attack effectiveness of hardware Trojans in a reconfigurable interconnection network. The experimental results demonstrate that the attack is highly effective, resulting in accuracy degradations of 90.91% for LeNet, 88.48% for AlexNet, and 88.82% for VGG. Next, the attack effectiveness of using 20-bit image data as the triggering condition is evaluated. The results indicate that the attack reduces accuracy by 12.23% for LeNet, 16.23% for AlexNet, and 12.75% for VGG. These accuracy degradations highlight the considerable attack effectiveness of the proposed hardware Trojan attack. By modifying the data path, the hardware Trojans lead to misconfiguration of the arithmetic circuit, thereby impacting the accuracy of the accelerator. Overall, the experimental findings demonstrate that the designed hardware Trojan attack has substantial attack effectiveness. The modifications made by the Trojans, which alter the data path, directly contribute to the misconfiguration of the arithmetic circuit, resulting in significant accuracy degradation in the system.

The overhead of hardware Trojans plays a crucial role in determining the feasibility of a Trojan attack. Trojans with high hardware overhead are more likely to be detected, so it is essential to keep the overhead as minimal as possible to avoid detection. In the context of this study, the trigger component and the payload component are the main contributors to the hardware costs of the hardware Trojan attacks. These components are separate from critical paths and consume logic hardware resources. The trigger component is often implemented using comparators, while the payload component is typically realized using XOR gates. As described in relation to the stealthiness evaluation, when using 24 bits of image data as the triggering condition, almost none of the original images from both datasets were able to activate the hardware Trojan payloads. Therefore, the trigger component in a hardware Trojan can be designed to have a specific bit sequence, typically consisting of 24 bits, representing the triggering condition. Table 5 presents the hardware overheads of the original accelerator (without Trojans) and the malicious accelerator (containing Trojans). The results indicate that the hardware overhead introduced by the Trojans is remarkably low, with an increase of only 0.27%. Such a minimal increase in resource consumption, primarily in terms of LUT (Look-Up Table) usage in an FPGA, suggests that the hardware Trojans are highly unlikely to be detected. This low overhead further reinforces the feasibility of hardware Trojan attacks against CNN accelerators.

### 4.3. Detection Effectiveness

In Section 3.2, the proposed countermeasure design is introduced regarding two cases involving single Trojans in one set and multiple Trojans in multiple sets. To conduct these experiments, configuration contexts are employed as challenges and fed into the PUF structure based on a matrix of multiplexers (MUXs) to obtain challenge–response pairs (CRPs). These CRPs are subsequently compared with the original CRP samples. The objective of these experiments is to assess the effectiveness of the proposed countermeasures in detecting and mitigating the presence of hardware Trojans. By utilizing configuration contexts as challenges and obtaining CRPs, the proposed countermeasure design can identify any discrepancies or abnormalities between the obtained CRPs and the original CRP samples. This comparison enables the detection of potential hardware Trojan attacks and facilitates the implementation of countermeasures to neutralize their effects. By conducting these evaluations and comparisons, the effectiveness of the proposed countermeasure design can be gauged in terms of its ability to identify and counteract hardware Trojan attacks based on the differences observed in the CRPs obtained from the PUF structured on the MUX matrix compared to the original CRP samples.

**Figure 13:**
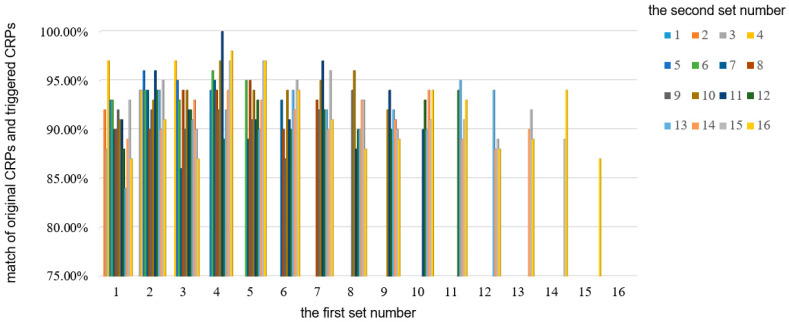
The comparison of original CRPs and triggered CRPs in the case of two hardware Trojans.

Table 6 presents the experimental results for the first case, where a Trojan is injected into the first set in advance. In this case, the enable signal is set to 1 to select output Q, and only one of the “choose” signals is set to 1 while the other signals are set to 0. This configuration allows the skipping of the set where “choose[i] = 1”. The results show that when the first set is skipped, the comparison of CRPs remains the same as the original CRPs, indicating that the delay path is not altered. However, for the remaining sets, the CRP comparison does not yield a 100% match. This indicates that the delay path is modified when these sets are skipped, suggesting the presence of a Trojan in those sets. By observing the differences in the CRP comparisons, it can be concluded that a hardware Trojan exists in the first set. In the second case, Figure 13 illustrates the comparison between the original CRPs and the triggered CRPs. In this experiment, any two sets are selected to be skipped, resulting in a total of 64 possible combinations. The *x*-axis in Figure 13 represents the first set number, while the bars represent the second set number that is skipped. For example, the red bar at the *x*-axis 1 represents the first and eighth skipping sets. Similar to the first case, the enable signal is set to 1, and two “choose” signals are set to 0 while the remaining signals are set to 1. When the fourth and eleventh sets are skipped (represented by the purple bar at the *x*-axis 4), the comparison between the original CRPs and the triggered CRPs shows no difference, indicating a 100% match. This result suggests that the delay path remains unchanged when these two sets are skipped, implying that the components in this delay path do not contain any Trojans. However, when other sets are skipped, the comparison results differ, indicating the presence of hardware Trojans in those specific sets.

The PUF consists of three main components: the delay path, enable logic, and arbiter. Table 7 shows the hardware overhead of the implemented based-PUF detection method. The implementation of a PUF in a digital circuit can consume 484 LUTs, resulting in a 0.57% increase in the size of the accelerator compared to the version without PUF. The resource utilization of the based-PUF detection methods is significantly low compared to the available resources, making them efficient applications in terms of defending CNN accelerators.

## 5. Discussion

This paper presents a study on hardware Trojan attacks in reconfigurable interconnection networks of FPGA-based CNN accelerators, followed by the proposal of a PUF-based countermeasure detection technique to mitigate these attacks. The hardware attack is evaluated based on its stealthiness, attack effectiveness, and hardware overhead. Additionally, the effectiveness of the countermeasure technique is tested to determine its ability to detect the presence and location of hardware Trojans. The experimental results highlight the impact of hardware Trojans on the accuracy of CNN accelerators, with significant accuracy degradations observed while incurring minimal hardware overhead. This showcases the potential threat posed by hardware Trojans in reconfigurable interconnection networks. Furthermore, the proposed countermeasure demonstrates promising capabilities in identifying the existence and specific locations of hardware Trojans. This detection technique provides a means to safeguard CNN accelerators against hardware attacks and enhance their security.

In future work, we intend to delve deeper into the study of various influences on CNN accelerators through different types of hardware Trojans. Additionally, we aim to explore and develop more countermeasures to fortify CNN accelerators against potential hardware attacks. In order to demonstrate that our research is based on a solid and reasonable experimental foundation, some popular open-source and publicly available accelerators will be incorporated into our experiments to further validate our findings, which can strengthen the credibility and reproducibility of the results. This ongoing research will contribute to the advancement of hardware security in the context of CNN accelerators.

## Figures and Tables

**Table 1:** Configuration words for different kernel sizes.

Kernel Size	MUX1	MUX2	MUX3	MUX4	MUX5	MUX6	MUX7
3 × 3	0	1	0	01	0	1	00
5 × 5	1	0	1	01	0	1	10
11 × 11	1	1	0	00	1	0	01

**Table 2:** Conditions of wrong connections caused by hardware Trojans.

Kernel Size	m1	m2	m3	m5	m6
3 × 3	S’2 = S2 + S1	S’2 = S2 − N3	S’2 = S2 − N3 − N4	S’4 = S4 + S3	S’4 = S4 − N7
5 × 5	S’1 = S1 − N1 − N2 − SP1	S’1 = S1	S’1 = S1 − N1 − N2 − SP1 − N3	S’2 = S2 − N5 − N6 − SP3	S’2 = S2
11 × 11	S’1 = S1 − N1 − N2 − SP1	S’1 = S1 − N1 − N2 − SP1 − N3	S’1 = S1 − N1 − N2 − SP1 − N3 − N4	S’1 = S1 − N1 − N2 − SP1 − N3 − N4 − SP2 − N5 − N6 − SP3	S’1 = S1

**Table 3:** The possible results for Trojans existing in two sets.

The State of Choose Signal	Comparison of CRPs
choose[1] = 1, choose[2] = 1	=
choose[1] = 1, choose[2] = 0	≠
choose[1] = 0, choose[2] = 1	≠
choose[1] = 0, choose[2] = 0	≠

**Table 4:** The accuracy of normal mode (no Trojan) and trigger modes (containing Trojans).

CNN Model	Accuracy		
	Normal Mode	Triggered Mode	
		Configuration Words	20-Bit Image Data
LeNet	98.21%	8.93%	86.20%
AlexNet	86.82%	10.00%	72.73%
VGG	90.26%	10.00%	78.75%

**Table 5:** The hardware overheads comparison between a clean accelerator (no Trojans) and a malicious accelerator (containing Trojans).

	Original Accelerator	Malicious Accelerator	Comparison
Overhead (LUT)	84.350 K	84.578 K	0.27% ↑ *

* ↑ means the increase.

**Table 6:** The comparison of CRPs for the case of a Trojan in one set.

Skipping Set Number	Match	Skipping Set Number	Match
1	100%	9	98%
2	97%	10	96%
3	98%	11	95%
4	95%	12	97%
5	95%	13	95%
6	98%	14	96%
7	91%	15	93%
8	97%	16	91%

**Table 7:** The hardware overheads comparison between accelerators with and without PUF.

	The Accelerator without PUF	The Accelerator with PUF	Comparison
Overhead (LUT)	84.350 K	84.834 K	0.57% ↑ *

* ↑ means the increase.

## Data Availability

Data are contained within the article.
